# Monitoring treatment of *Taenia solium*- neurocysticercosis by detection of circulating antigens: a case report

**DOI:** 10.1186/s12883-019-1282-x

**Published:** 2019-04-03

**Authors:** Richar Rodríguez-Hidalgo, Arturo Carpio, Erwin Van den Enden, Washington Benítez-Ortiz

**Affiliations:** 1 Instituto de Investigación en Salud Pública y Zoonosis, Universidad Central del Ecuador, Quito, Ecuador; 2 Facultad de Medicina Veterinaria y Zootecnia, Universidad Central del Ecuador, Quito, Ecuador; 3 Facultad de Ciencias Médicas, Universidad de Cuenca, Cuenca, Ecuador; 4 G.H. Sergievsky Center, Columbia University, New York, USA; 5 Department of Clinical Sciences, Institute of Tropical Medicine, Antwerp, Belgium

**Keywords:** Neurocysticercosis, *Taenia solium*, Treatment, Albendazole, Praziquantel

## Abstract

**Background:**

Parenchymal neurocysticercosis is a frequent cause of seizures in areas endemic for *Taenia solium.* At present there is scarce data on the evolution of the levels of circulating metacestodal antigen before, during and after treatment with anthelmintic drugs.

**Case presentation:**

A patient with paucisymptomatic neurocysticercosis (NCC) diagnosed by Ag-ELISA, and confirmed by MRI images, was treated with praziquantel, albendazole and dexamethasone. The level of circulating *T. solium* antigen was determined weekly. Circulating antigen disappeared from his blood within 14 days after the start of the treatment and correlated with the involution of the cysticerci in the brain shown by imaging. Seventeen years later, the patient has not shown any side effect nor symptoms related to the treatment or to NCC.

**Conclusions:**

If this encouraging finding is confirmed in a larger series of patients, this technique could be used to determine parasitological cure after treatment and might complement or sometimes replace sequential MRI-imaging of the brain.

## Background

Neurocysticercosis is a frequent cause of seizures in areas endemic for *Taenia solium*, the pig tapeworm, but the infection can be asymptomatic. The treatment of this condition is controversial i.a. [1–3]. One has to balance the risks that treatment with praziquantel and/or albendazole can lead to leakage of parasite antigen with an increase in perilesional edema. The possible triggering of neurological symptoms versus the risk of neurological problems due to growing cysts in the brain is always a risk. At present, neuroimaging studies (computed tomography and nuclear magnetic resonance) are the main tools for diagnosing NCC, immunological tests are also useful for confirming diagnosis [4]. Detection of circulating parasitic antigens in serum by Ag-ELISA with monoclonal antibodies has been used in clinical and field studies. The sensitivity of this test in serum for parenchymal NC ranges from 72 to 86% [5]. However, the main advantage of antigen detection is its specificity for diagnosis of multiple parasites and for monitoring the decrease in parasite burden in response to antiparasitic treatment [6]. This is why it has been suggested that Ag-ELISA can be of added value for diagnosis and treatment of NCC [7].

Since a number of years, detection of circulating *T. solium* antigen has become a possibility. The data on how the circulating antigen evolves under treatment is scarce [5, 6, 8–11]. We had the opportunity of studying this evolution in detail.

## Case presentation

**Fig. 1:**
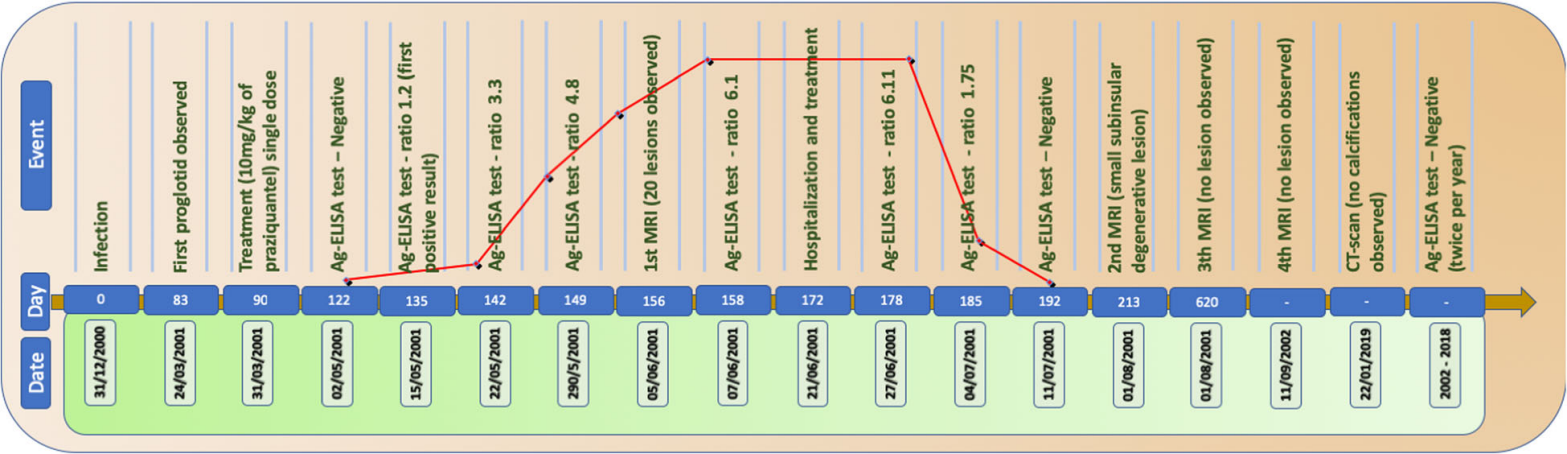
Timeline of the patient: Event flow and treatment monitoring and Antigen detecting enzyme linked immunosorbent assay (Ag-ELISA) assessment. Considering the day of infection as day 0, the first proglottids were observed in the stools on day 83. Tissue invasion probably occurred between day 83 and 90. A single dose praziquantel was given on day 90. Treatment for neurocysticercosis was given from day 172 till day 180. The Ag-ELISA results remained negative until day 135 when a slightly positive ratio of 1.2 was noted. This ratio gradually increased (i.e. 3.3 at day 142; 4.8 at day 149; 6.1 at day 158 and at day 178). On day 185 (i.e. five days after the end of treatment) there was a sharp decrease in circulating antigen (1.75). Since day 192 to year 2018 the test remained negative, i.e. no ratios higher than 1 were observed. Additionally, two MRI images were conducted in 2002 and 2008 without any evidence of lesions. Red line: Circulating antigen of *Taenia solium* metacestodes detected by Ag-ELISA, before and after anthelmintic treatment. Ratio = OD sample/OD cut-off (OD = Optical Density). Positivity starts at ratio-values above 1

A 27-year-old male Ecuadorian observed taeniid proglottids in his stools on March 24, 2001. The date of infection was precisely known as he remembered having consumed undercooked pork in the Northern Andes on a single occasion, on December 31, 2000. This day will be regarded as day 0 (Fig. 1). He stated that he never had tapeworm infections before. At the veterinary department of the “Universidad Central del Ecuador” these proglottids were identified as *T. solium*. Elimination of proglottids went on for one week. Proglottids were carefully collected and stored in Ethanol 70%.

On day 90 a single oral dose of praziquantel 10 mg/kg body weight was administered together with 5 mg of Bisacodilo (Dulcolax®) at night. Faeces were collected in a hermetic plastic bag and transported to the lab. Around 1,5 m of an expulsed tapeworm with an intact scolex was recovered*.* Review of the adult cestode allowed to identify it as a *T. solium* specimen which was confirmed by morphology, isoenzyme electrophoresis based on glucose phosphate isomerase (GPI-zymograms) and molecular protocols as were described by Rodríguez-Hidalgo et al. [12].

**Fig. 2:**
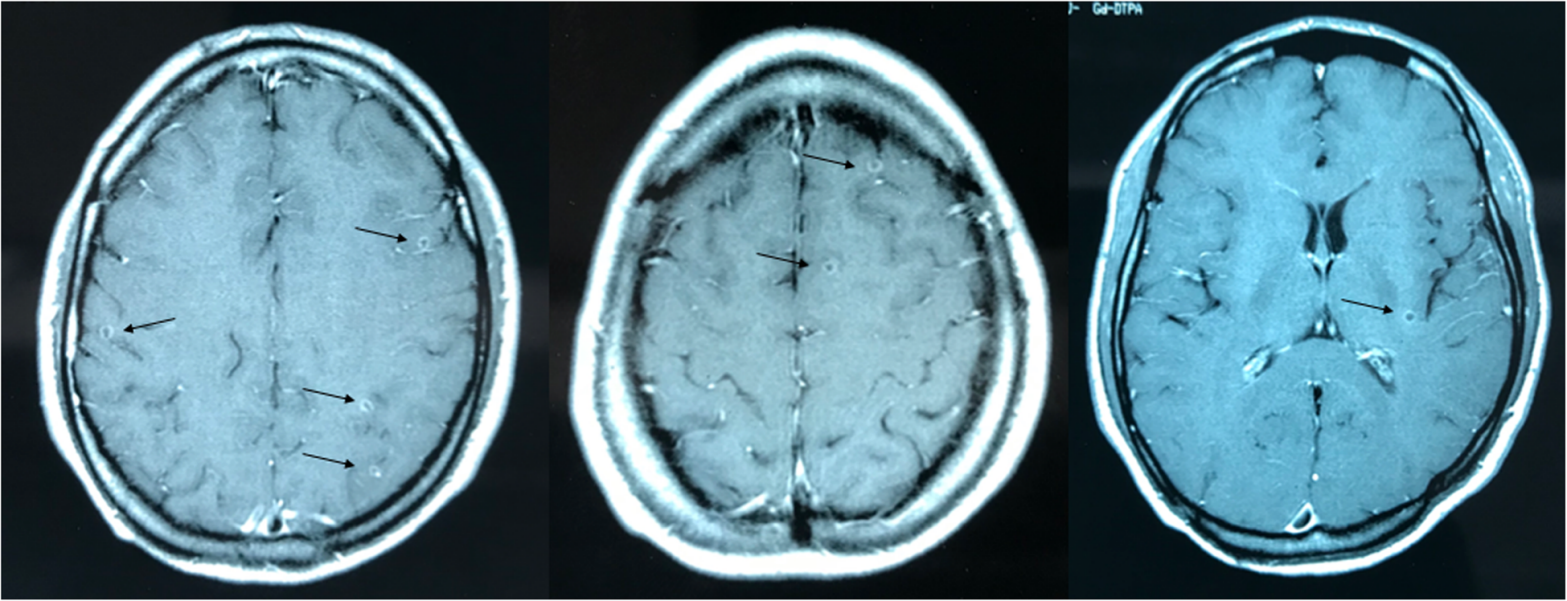
Brain magnetic resonance imaging (MRI) showed some cysticerci in the supra- and infratentorial brain parenchyma (First MRI image conducted in 5/6/2001)

As a precaution, the patient was advised to have a check-up for cysticercosis. A B158/B60 monoclonal antibody-based sandwich ELISA was used for detecting of circulating cysticercal antigen in serum (Ag-ELISA) [13, 14], whereby the cut-off level is calculated by comparing the optical density of each sample with the mean of a series of eight negative human serum samples at a probability level of *P* = 0.001. The results are expressed as a ratio, i.e. values higher than one, considered as being positive [13–16]. Initially, this assay has been developed for the ante-mortem diagnosis of bovine cysticercosis, [14, 17], later-on Ag-ELISA usefulness for the diagnosis of human cysticercosis has been tested [7, 18, 19]. The Ag-ELISA performed on day 122 was negative. The patient, in the meantime developing a slight headache and living in Belgium, was referred to the outpatient department, Institute of Tropical Medicine, Antwerp. A physical examination, full blood count and biochemical profile were normal and stools for ova were negative. The retinae did not show any lesion or cysticerci. An Ag-ELISA performed on day 135 was weakly positive. The test became clearly positive on day 142. A magnetic resonance image (MRI) of the brain was taken on day 156, revealing 20 cysticerci in the supra- and infratentorial brain parenchyma (Fig. 2). The lesions were small (3–5 mm), in degenerative phase (granular-nodular). There were no mass effects, intraventricular cysts or signs of hydrocephalus.

**Fig. 3:**
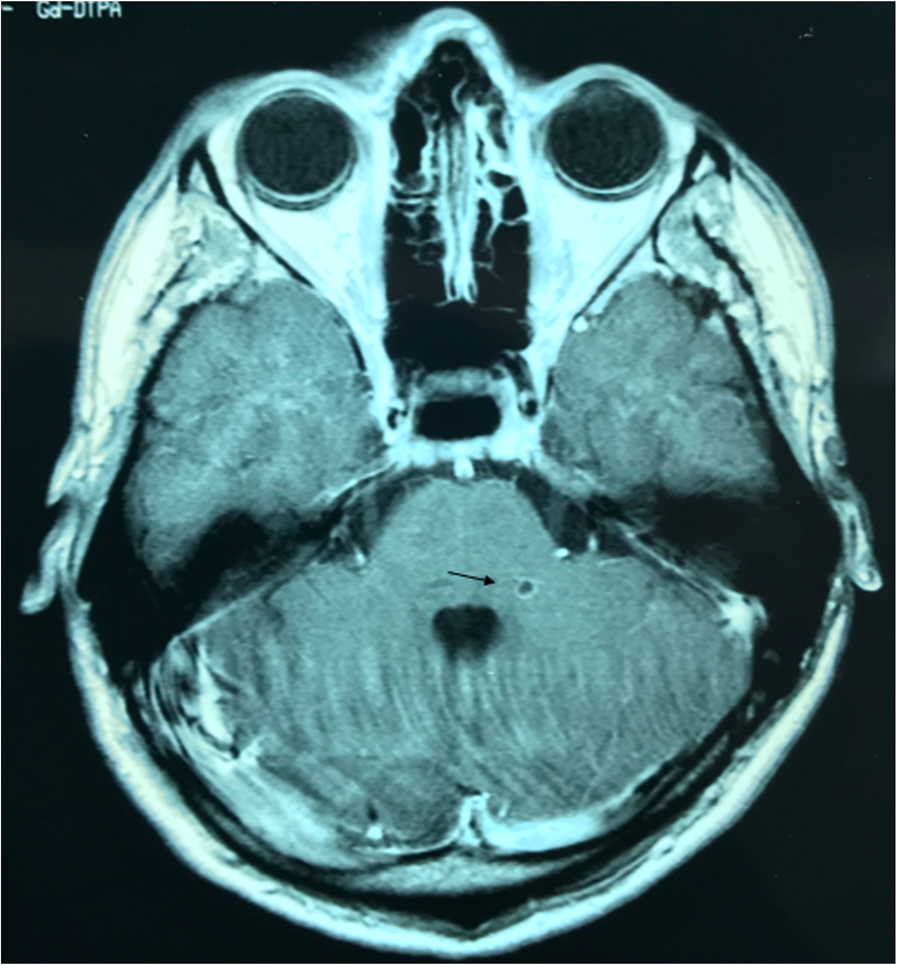
Brain magnetic resonance imaging (MRI) showed one small subinsular degenerative lesion after treatment (Second MRI image conducted in 1/8/2001)

The patient was hospitalised (the standard informed consent of the hospital was signed by the patient) and treatment with dexamethasone (tapering dose), praziquantel 3 g single dose, followed by albendazole 800 mg per day for 8 days was administered (day 172 till day 180) proposed by the physician. A second MRI image, taken on day 213 showed the near disappearance of cysticerci in the brain, with the exception of one small subinsular degenerative lesion (Fig. 3). The patient did not experience any particular symptom during treatment, but shortly after this treatment a slight temporary hepatitis was observed (blood biochemical profile), contributed to a secondary reaction to the medication. Back in Ecuador, the patient was follow-up by a physician who advised Ag-ELISA detection twice per year which were negative. In addition, two MRI and one Computed tomography scan (CT-scan) were conducted in 2002, 2008 and 2019, respectively; no lesions or calcifications were observed in these images. Seventeen years later, the patient has not shown any side effects nor symptoms related to the treatment or to NCC. No Taeniosis or NCC reinfection were also reported. The treatment was successful.

## Discussion and conclusion

Carriers of adult *T. solium* have a high risk for neurocysticercosis not only for themselves but their household members and even the community. The present case of cysticercosis is most likely a consequence of external faeco-oral self-contamination or an internal autoinfection. Proglottids can be vomited [20] and passage through the stomach can activate the eggs. The possible role herein of the taenicidal treatment with single-dose praziquantel remains hypothetical. The date of infection (eating contaminated pork) was precisely known. The onset of the dissemination of oncospheres and development of metacestodes, and subsequently the age of the cysticercosis can be estimated to have happened in the week after the first proglottids were noticed or around the time of the initial single dose treatment was taken. One can argue that another asymptomatic cysticercosis could have been present before December 31, but this is unlikely. Since the period that gravid proglottids were produced was short, it can therefore be assumed that the Ag-ELISA allowed for the detection of metacestodes even before being fully developed. It corresponds with observations on bovine cysticercosis [13, 14]. The antigen levels became positive about 7–8 weeks after the presumed dissemination of *T. solium* oncospheres. This case report highlights (a) a typical incubation period for intestinal *T. solium* infection of 83 days, (b) the need for being alert for the possibility of neurocysticercosis in carriers of intestinal *T. solium* from endemic areas*,* even when treated with a single dose praziquantel, (c) the time lag of about 7 weeks between activation of oncospheres and seroconversion by Ag-ELISA, (d) the negativation of Ag-ELISA within 2 weeks of the start of treatment corresponding with cure as assessed by MRI-imaging of the brain, (e) a second screening at least two months after anthelmintic treatment of intestinal taeniosis is warranted, (f) repeated assays for circulating antigen were done twice per year until 2018 to monitor the evolution of an infection with larval *T. solium* and the efficacy of treatment; all tests have been negative. The Ag-ELISA has the added advantage that the relation between positive results and the presence of living larvae is closer than by antibody detecting assays [7, 18, 19]. If these encouraging findings would be confirmed in a larger series, the use of Ag-ELISA for the detection of live cysticerci would be a valuable complement or possible alternative for MRI in cases where MRI would be too costly. In spite of the fact that the monoclonal antibodies were raised against metabolic antigens of *T. saginata* metacestodes, they crossreact with other *Taenia* spp. i.a. *T. solium* [7, 18, 19]. It remains however restricted to the genus *Taenia* [13] and to the larval stages.

## Abbreviations

Ag-ELISA: Antigen detecting enzyme linked immunosorbent assay
CT-Scan: Compute tomography scan
GPI: Glucose Phosphate Isomerase
MRI: Magnetic Resonance Imaging
NCC: Neurocysticercosis
PCR: Polymerase Chain Reaction
RFLP: Restriction Fragment Length Polymorphism
